# Development of Microsatellite Markers in the Branched Broomrape *Phelipanche ramosa* L. (Pomel) and Evidence for Host-Associated Genetic Divergence

**DOI:** 10.3390/ijms15010994

**Published:** 2014-01-13

**Authors:** Valérie Le Corre, Carole Reibel, Stéphanie Gibot-Leclerc

**Affiliations:** 1 INRA, UMR1347 Agroécologie, Dijon F-21000, France; 2 Agrosup-Dijon, UMR1347 Agroécologie, Dijon F-21000, France; E-Mails: carole.reibel@agrosupdijon.fr (C.R.); stephanie.gibot-leclerc@dijon.inra.fr (S.G.-L.)

**Keywords:** *Phelipanche ramosa*, parasitic plant, microsatellite markers, genetic diversity, host races

## Abstract

*Phelipanche ramosa* is a parasitic plant that infects numerous crops worldwide. In Western Europe it recently expanded to a new host crop, oilseed rape, in which it can cause severe yield losses. We developed 13 microsatellite markers for *P. ramosa* using next-generation 454 sequencing data. The polymorphism at each locus was assessed in a sample of 96 individuals collected in France within 6 fields cultivated with tobacco, hemp or oilseed rape. Two loci were monomorphic. At the other 11 loci, the number of alleles and the expected heterozygosity ranged from 3 to 6 and from 0.31 to 0.60, respectively. Genetic diversity within each cultivated field was very low. The host crop from which individuals were collected was the key factor structuring genetic variation. Individuals collected on oilseed rape were strongly differentiated from individuals collected on hemp or tobacco, which suggests that *P. ramosa* infecting oilseed rape forms a genetically diverged race. The microsatellites we developed will be useful for population genetics studies and for elucidating host-associated genetic divergence in *P. ramosa*.

## 1. Introduction

Among flowering plants, approximately 3000 species (1%) are parasitic. These parasitic plants form a close connection with the vascular system of their host plant through a specialized organ, the haustorium, through which they remove water, mineral salts, and carbon elements [1,2]. Broomrapes are chlorophyll-lacking, obligate root parasitic plants. Among broomrapes, branched broomrape, *Phelipanche ramosa* (L.) Pomel (syn. *Orobanche ramosa* L. Pomel), is the most widespread species. It occurs throughout the South-East of Europe, West Asia and North Africa and was accidentally introduced in several other parts of the worlds (e.g., in Australia, the USA and Chile). It is a noxious weed that infects numerous crops, among which tobacco, tomato and hemp, particularly in countries surrounding the Mediterranean basin and in Central Europe [3,4]. From the 1980s, infections have been identified on a new host crop: oilseed rape, in several European countries such as Bulgaria, France, Italy and Spain [3,5]. In western France, a massive extension of *P. ramosa* to oilseed rape fields has been observed since the beginning of the 1990s [6,7], causing heavy yield losses. This crop has now become the primary host for the parasite, along with tobacco, hemp and buckwheat [5,8].

Investigations of the extent of genetic variation in *P. ramosa* have been fairly limited up to now. All previous studies used dominant markers, either RAPD [8,9], ISSR [5] or AFLP [10]. Their results suggested the existence of two or three diverged genetic groups among populations of *P. ramosa*, which might be associated with host specificity. Among the molecular markers available to study genetic variation in natural plant populations, microsatellites or SSRs (simple sequence repeats) are especially valuable because of their high reproducibility, high degree of polymorphism and codominant inheritance. Despite the economic importance of broomrapes, up to now SSRs have been developed for only one species, the sunflower broomrape *Orobanche cumana* [11]. Here, we report the development of microsatellite markers for *P. ramosa* and investigate whether these markers reveal some genetic divergence in relation with the host crop, and especially with the ability to infect oilseed rape.

## 2. Results and Discussion

### 2.1. Development of Microsatellite Markers

The 454-sequencing data retrieved from the Sequence Read Archive (SRA) contained a total of 1,516,538 reads, with an average read length of 563 bp. After quality filtering, we retained a reduced set of 172,364 reads (11%), with a mean length of 395 bp (range: 250–483 bp). After discarding sequences without microsatellite motifs using the QDD software, the remaining sequences were filtered for redundancy and multiple copies in the sequence data set. In total, 2091 microsatellite loci were identified, and automated primer design was successful for 425 loci.

We selected 63 primers pairs with highest quality based on a low self-end complementarity and high end-stability of the primers. PCR amplification was assayed using 6 DNA samples. After these initial PCR assays, 13 microsatellites markers reliably yielding a single PCR product were retained. Based on Sanger sequencing, these markers were all confirmed to contain the expected microsatellite motif.

### 2.2. Genotyping and Population Genetics Analysis

Polymorphism at each of the 13 developed microsatellites was determined using 96 *P. ramosa* individuals from six populations. Among the 13 loci, 2 were found to be monomorphic (Table 1). As the samples considered here were all collected in France, and therefore only represent a small subset of the wide distribution area of *P. ramosa*, it is possible that these 2 microsatellite markers would be found polymorphic using a geographically enlarged sampling scheme. Therefore we consider them as potentially useful for future studies of the genetic variation in *P. ramosa*. For the remaining eleven markers, the number of alleles per locus ranged from two to six, the observed heterozygosity (*H*_o_) ranged from 0.000 to 0.025 and the expected heterozygosity (*H*_e_) ranged from 0.308 to 0.601 (Table 1). The observed heterozygosity over all loci and plants was very low (0.003), and the overall mean value of the inbreeding coefficient *Fis* was 0.863. These figures are well in agreement with the assumption that *P. ramosa* is a self-fertile species with a very high rate of selfing [3].

Genetic variation within each of the studied populations was very low: in each population, only one, two or three markers among the eleven ones were polymorphic (Table 2). Moreover, for each microsatellite that was polymorphic within a population, the frequency of the most frequent allele was always high (between 0.594 and 0.969, mean = 0.855) and minor alleles generally differed from the most frequent allele by a small number of repeat motifs.

The host crop from which the populations were collected was the key factor structuring the genetic variation at microsatellites. The four populations collected from oilseed rape fields shared the same fixed or major alleles at all loci (Table 2). At six loci (Phera14, Phera18, Phera20, Phera40, Phera46 and Phera53), the two populations collected on tobacco and hemp shared a same allele, which was different from alleles found in the four populations collected on oilseed rape. At locus Phera19, two alleles were present, one that characterized the tobacco population and one that was shared between the hemp population and the four oilseed rape populations. At the other loci, three distinct major alleles characterized each of the three host crops.

**Figure 1.**
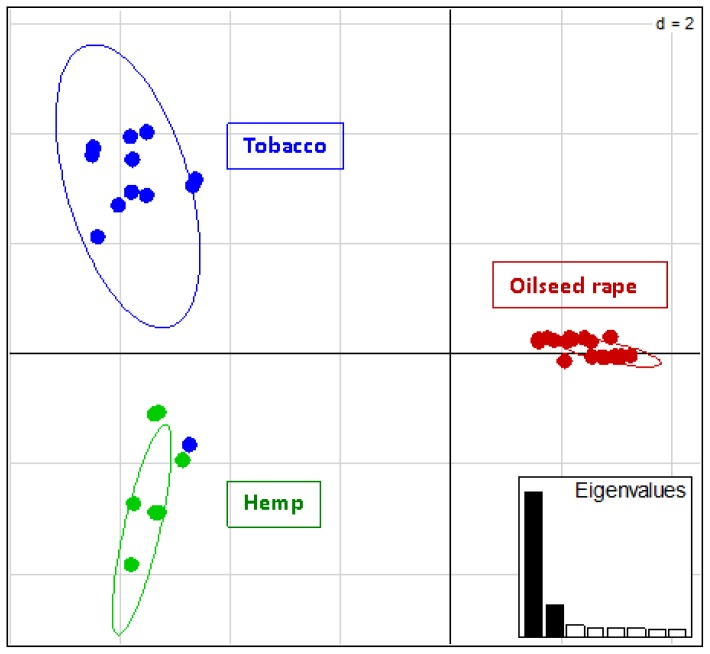
Genetic variation detected at eleven polymorphic microsatellites in 96 *P. ramosa* individuals sampled from six populations: projection of individual data on the first and second axes of a Principal Component Analysis. The host crops on which individuals were collected are represented as different colors.

Polymorphic microsatellites displayed 8 different multilocus genotypes in the population collected on tobacco, four multilocus genotypes in the population collected on hemp and five multilocus genotypes in the four populations collected on oilseed rape. None of those multilocus genotypes was shared between different host crops. In tobacco, two genotypes were predominant (frequencies of 0.36 and 0.21), while one single genotype was predominant in hemp (frequency 0.79), and one single genotype was also predominant in oilseed rape (frequency 0.82). Finally, a multivariate analysis of the genetic variation at the eleven polymorphic microsatellites perfectly distinguished among *P. ramosa* individuals collected on different host crops (Figure 1). The first principal component accounted for 56% of the variation and differentiated individuals collected on oilseed rape from all other individuals, while the second principal component accounted for 12% of the variation and differentiated individuals collected on tobacco from those collected on hemp, at the exception of one individual collected on tobacco that seemed to be a hybrid between these two genetic groups.

Geographic effects might have contributed to the genetic structure observed. However, this seemed unlikely here. Population PO (hemp) was localized in the north-east of France, more than 400 km away from the other five populations, which were all localized in the same area (West of France), at less than 50 km from one another (Table 3). Thus geographic structure did not match with genetic structure.

## 3. Experimental Section

### 3.1. Plant Material and DNA Extraction

Parasite seeds were collected from French natural populations of *P. ramosa* that had severely infected hemp, oilseed rape and tobacco fields in 2001 and 2002. The plants sampled were distributed throughout each cultivated field. Geographic origins of the populations are shown in Table 3. The total genomic DNA of 96 individuals (16 from each of the six populations) was extracted using a rapid method based on incubation at high temperature in a TrisHCl-EDTA buffer [12].

### 3.2. Isolation of Microsatellite Markers

Raw sequence data for the *P. ramosa* genome was retrieved from the NCBI Sequence Read Archive (http://www.ncbi.nlm.nih.gov/sra). The experiment was reported in [13]. It consisted in one run of sequencing on one GS Titanium PicoTiterPlate on a 454 Genome Sequencer (Roche Diagnostics, Indianapolis, IN, USA), using the recommended standard protocols and chemistry.

After converting raw sequence data to the fastq format, quality control and filtering was performed using the PRINSEQ web (http://edwards.sdsu.edu/prinseq) [14]. Briefly, read ends were first trimmed by quality scores, after which only sequences longer than 250 bp, having a mean Phred quality score higher than 35% and less than 1% Ns were retained. Exact sequence duplicates were removed.

Microsatellites were identified by running the QDD pipe-line [15] using the following criteria: a minimum of eight repeats for dinucleotide motifs, six repeats for trinucleotide motifs and five repeats for tetranucleotide motifs and a minimum length of 100 pb for the PCR product. Primer sequences generated by QDD were checked for end stability, self-complementarity and complementarity between primers in a same pair to avoid the formation of primer dimers.

### 3.3. DNA Amplification and Genotyping

PCR amplification was first assayed on a subsample of 6 individuals. PCR amplifications were performed using a Mastercycler (Eppendorf, Hamburg, Germany) thermocycler, in a 20 μL reaction mix containing 70 mM Tris-HCL, 2 mM MgCl_2_, 17 mM (NH_4_)_2_SO_4_, 10 mM β-mercaptoethanol, 0.05% (*w*/*v*) polyoxyethylene-ether W1, 0.2 mg/mL bovine serum albumin, 200 mM of each dNTP, 10 ng genomic DNA, 0.5 units of Taq DNA polymerase, and 0.2 μM each of reverse and forward primers. The PCR program used consisted of 5 min at 95 °C, followed by 37 cycles of 5 s at 95 °C, 10 s at 60 °C and 30 s at 72 °C. Amplicons were visualised under UV light by electrophoresis on a 3% (*w*/*v*) agarose gel stained with ethidium bromide. When a single, intense amplicon was obtained from all 6 plants, it was sequenced on both strands to confirm the presence of the expected microsatellite motif.

For genotyping, the DNA extracts of all plants were diluted 50-fold prior to genotyping with fluorescent dye-labelled markers (6-FAM, NED, VIC, PET). PCR products were assayed on an ABI 3730XL sequencer (Applied Biosystems, Foster City, CA, USA) using GeneScan 500 LIZ dye size standard (Applied Biosystems). Amplicon sizes were analyzed with the software Peak Scanner 1.0 (Applied Biosystems).

### 3.4. Data Analysis

The number of alleles (*N*_a_) per locus, observed heterozygosity (*H*_o_), expected heterozygosity (*H*_e_), and inbreeding coefficient (*Fis*) were estimated using GenAlex 6.5 [16]. Overall and per-population values were calculated. To summarize the genetic variation among individuals, a multivariate analysis was performed via Principal Component Analysis, using the R package adegenet [17].

## 4. Conclusions

We report the development and characterization of 13 microsatellites markers in the branched broomrape, *Phelipanche ramosa*. Eleven out of the 13 markers were polymorphic on a set of 96 individuals of *P. ramosa* sampled in France. The level of polymorphism was low, especially within populations. This low level of variation may be explained by a high selfing rate in this species and possibly by bottlenecks associated with founding events for the studied populations. Genetic variation was strongly structured by the three host crops on which individuals were collected: tobacco, hemp and oilseed rape. Our results suggest that *P. ramosa* infecting oilseed rape in France belong to a recently evolved but highly genetically divergent host race. Analyzing more populations collected from different hosts will be necessary to confirm this. The microsatellites developed here will be useful for future population genetics studies in *P. ramosa* and for elucidating the presence of genetically diverged host races.

## Figures and Tables

**Table 1.** The set of 13 microsatellite markers developed in *P. ramosa*: locus name, repeat motif, primer sequences, annealing temperature (*T*_a_), allele size range, number of alleles (*N*_a_), observed (*H*_o_) and expected (*H*_e_) heterozygosities and GenBank accession number.

Locus	Repeat motif	Primer sequences (5′-3′)	*T*_a_ (°C)	Size range (bp)	*N*_a_	*H*_o_	*H*_e_	GenBank accession No.
Phera02	(AT)_4_T(AT)_15_	F: CTCACACCCGGACAAAGTTC R: GGTCAATCCAGACTCGCTTC	60	118–143	5	0.013	0.542	SRR364824.1090
Phera10	(AT)_12_	F: CCACATCAGCAACAATAGCAA R: CATGATCTTTGATAGGCACGA	60	116–144	6	0.011	0.522	SRR364824.897002
Phera14	(CTT)_12_	F: ATGGGAACGTCTTATGGCAC R: GTGGATCCCATGGTCTTTTG	60	330–336	2	0.000	0.442	SRR364824.865553
Phera18	(GAT)_10_	F: TGGTGAAGAAACGAACAATCA R: CATTCTCCACATTTTCCGCT	60	122–131	2	0.000	0.481	SRR364824.939350
Phera19	(ACC)_9_	F: AGCAGTTACGGAACCACCAC R: CAGTTTCACGGGGAGAATGT	60	140–143	2	0.000	0.308	SRR364824.843255
Phera20	(ATT)_9_	F: TCACAATACGCTCCAAGCTG R: GAGTTCACGTTGTGGGCTTT	60	134–159	2	0.000	0.484	SRR364824.981601
Phera28	(AT)_5_A(AT)_9_	F: TGCGTCTGTTCTCTCAACGA R:CGGAAAGGAAGGTTCTTATTTG	60	195–197	2	0.000	0.432	SRR364824.879658
Phera38	(AT)_11_	F: TCAGGTGAAGTGTTGCAATTAT R: GCCTTTCTTGTTCCTTGTCG	60	243–261	6	0.000	0.601	SRR364824.320858
Phera40	(AC)_11_	F: CATCTGAGGTGCACATTACGTC R: TTTGTCATTGCTTCGTGGAG	60	199–203	2	0.000	0.442	SRR364824.873170
Phera46	(CAT)_8_(CAA)(CAT)_3_	F: GTCACTTGTCCCAACATCCC R: TCTCGCCTCGCTGATAAAAT	60	237–258	3	0.000	0.446	SRR364824.286970
Phera47	(ACC)_8_	F: GCATCAGCATCAGAAGACGA R: GGTGGTTGGTAGTTGGTTGG	60	139	1	0.000	0.000	SRR364824.375211
Phera53	(ATC)_2_(ATT)(ATC)_8_	F: CAACATCAGCGTCAACCATC R: GATCACCGACTGATGGAGGT	60	128–134	3	0.011	0.449	SRR364824.227061
Phera59	(TTA)_7_	F: CGTTGGCCTTAAATCGCTAC R: TTGGTTTTGTGTGTGGAGGA	60	304	1	0.000	0.000	SRR364824.1022037

**Table 2.** Genetic variation at 11 polymorphic microsatellites in six populations of *P. ramosa*. The crop from which each population was sampled is indicated within parentheses.

		PR (Tobacco)				PO (Hemp)				GI (Oilseed rape)		
		
Locus	*N*_a_	Allele sizes	*H*_o_	*H*_e_	*N*_a_	Allele sizes	*H*_o_	*H*_e_	*N*_a_	Allele sizes	*H*_o_	*H*_e_
			
Phera02	3	131/135/137	0.000	0.338	3	135/137/143	0.071	0.196	1	118	0.000	0.000
Phera10	5	136/138/140/142/144	0.063	0.592	2	138/140	0.000	0.219	1	116	0.000	0.000
Phera14	1	330	0.000	0.000	1	330	0.000	0.000	1	336	0.000	0.000
Phera18	1	122	0.000	0.000	1	122	0.000	0.000	1	131	0.000	0.000
Phera19	1	140	0.000	0.000	1	143	0.000	0.000	1	143	0.000	0.000
Phera20	1	134	0.000	0.000	1	134	0.000	0.000	1	159	0.000	0.000
Phera28	2	195/197	0.000	0.133	1	195	0.000	0.000	1	197	0.000	0.000
Phera38	1	243	0.000	0.000	1	247	0.000	0.000	2	257/259	0.000	0.142
Phera40	1	203	0.000	0.000	1	203	0.000	0.000	1	199	0.000	0.000
Phera46	1	237	0.000	0.000	1	237	0.000	0.000	1	258	0.000	0.000
Phera53	1	134	0.000	0.000	1	134	0.000	0.000	1	131	0.000	0.000

		**SJ (Oilseed rape)**				**SA (Oilseed rape)**				**SV (Oilseed rape)**		
			
**Locus**	***N*_a_**	**Allele sizes**	***H*_o_**	***H*_e_**	***N*_a_**	**Allele sizes**	***H*_o_**	***H*_e_**	***N*_a_**	**Allele sizes**	***H*_o_**	***H*_e_**
			
Phera02	1	118	0.000	0.000	1	118	0.000	0.000	1	118	0.000	0.000
Phera10	1	116	0.000	0.000	1	116	0.000	0.000	1	116	0.000	0.000
Phera14	1	336	0.000	0.000	1	336	0.000	0.000	1	336	0.000	0.000
Phera18	1	131	0.000	0.000	1	131	0.000	0.000	1	131	0.000	0.000
Phera19	1	143	0.000	0.000	1	143	0.000	0.000	1	143	0.000	0.000
Phera20	1	159	0.000	0.000	1	159	0.000	0.000	1	159	0.000	0.000
Phera28	1	197	0.000	0.000	1	197	0.000	0.000	1	197	0.000	0.000
Phera38	2	259/261	0.000	0.271	4	255/257/259/261	0.000	0.459	2	257/259	0.000	0.133
Phera40	1	199	0.000	0.000	1	199	0.000	0.000	1	199	0.000	0.000
Phera46	2	255/258	0.000	0.121	1	258	0.000	0.000	1	258	0.000	0.000
Phera53	1	131	0.000	0.000	1	131	0.000	0.000	2	128/131	0.063	0.061

PR, PO, GI, SJ, SA and SV are abbreviated names of the populations (see Table 3); *N*_a_: number of alleles; *H*_o_: observed heterozygosity; *H*_e_: expected heterozygosity.

**Table 3.** Geographic origins of the six *P. ramosa* populations used in this study.

Name	Localization	Latitude (N)	Longitude (E)	Crop
PR	Priaires	46.143	−0.607	Tobacco
PO	Pont-sur-Seine	48.519	3.595	Hemp
GI	Gibourne	45.935	−0.312	Oilseed rape
SJ	Saint-Jean-d’Angély	45.946	−0.519	Oilseed rape
SA	Savarit	46.1142	−0.8302	Oilseed rape
SV	Savarit	46.1142	−0.8302	Oilseed rape
